# Scanning Electron Microscopy Imaging of Large DNA Molecules Using a Metal‐Free Electro‐Stain Composed of DNA‐Binding Proteins and Synthetic Polymers

**DOI:** 10.1002/advs.202309702

**Published:** 2024-05-05

**Authors:** Chanyoung Noh, Yoonjung Kang, Sujung Heo, Taesoo Kim, Hayeon Kim, Junhyuck Chang, Priyannth Ramasami Sundharbaabu, Sanghee Shim, Kwang‐il Lim, Jung Heon Lee, Kyubong Jo

**Affiliations:** ^1^ Department of Chemistry Sogang University Seoul 04107 South Korea; ^2^ School of Advanced Materials Science and Engineering Department of MetaBioHealth Sungkyunkwan University (SKKU) Suwon 16419 South Korea; ^3^ Department of Chemistry Korea University Seoul 02841 South Korea; ^4^ Department of Chemical and Biological Engineering Sookmyung Women's University Seoul 04312 South Korea

**Keywords:** DNA binding protein, polyvinylpyrrolidone, quantum dot labeling, SEM DNA imaging, SEM optical DNA mapping

## Abstract

This paper presents the first scanning electron microscopy (SEM)‐based DNA imaging in biological samples. This novel approach incorporates a metal‐free electro‐stain reagent, formulated by combining DNA‐binding proteins and synthetic polymers to enhance the visibility of 2‐nm‐thick DNA under SEM. Notably, DNA molecules stain with proteins and polymers appear as dark lines under SEM. The resulting DNA images exhibit a thickness of 15.0±4.0 nm. As SEM is the primary platform, it integrates seamlessly with various chemically functionalized large surfaces with the aid of microfluidic devices. The approach allows high‐resolution imaging of various DNA structures including linear, circular, single‐stranded DNA and RNA, originating from nuclear and mitochondrial genomes. Furthermore, quantum dots are successfully visualized as bright labels that are sequence‐specifically incorporated into DNA molecules, which highlights the potential for SEM‐based optical DNA mapping. In conclusion, DNA imaging using SEM with the novel electro‐stain offers electron microscopic resolution with the ease of optical microscopy.

## Graphical abstract

This work demonstrates SEM‐based DNA imaging using a metal‐free electro‐stain reagent. It enhances the visibility of 2‐nm‐thick DNA as a dark line. DNA images exhibit 15.0 ± 4.0 nm thickness, enabling high‐resolution imaging of various DNA structures. Quantum dots, serving as bright labels on DNA, are successfully visualized, suggesting potential for SEM‐based optical DNA mapping, combining electron microscopic resolution with optical microscopy ease.

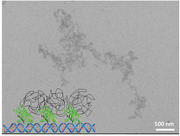

## 1 Introduction

DNA imaging can capture images of DNA and its associated biomolecules, thereby providing intricate molecular details regarding molecular structure and function. Since the first single‐molecule DNA visualization in 1948,^[^ ^1^ ^]^ transmission electron microscopy (TEM) has played a crucial role in the observation of DNA,^[^ ^2^ ^]^ chromatin,^[^ ^3^ ^]^ and protein‐DNA complexes.^[^ ^4^ ^]^ To overcome the low electron scattering properties of DNA, heavy metal salts, such as uranyl acetate, have been utilized for DNA staining in combination with the shadow casting method.^[^ ^5^ ^]^ Without metal staining, cryo‐electron microscopy (cryo‐EM) has been used to visualize DNA and its complexes, achieving a resolution of 2 nm across the width of double‐stranded DNA molecules.^[^ ^6^ ^]^ Besides TEM, other DNA imaging methods that are widely utilized include fluorescence microscopy,^[^ ^7^ ^]^ super‐resolution microscopy,^[^ ^8^ ^]^ and atomic force microscopy (AFM).^[^ ^9^ ^]^

Currently, fluorescence‐based DNA imaging is the primary method for visualizing large DNA molecules and widely used due to its simplicity, accessibility, and capability for live imaging despite the intrinsic limitation of low resolution. Although various innovative high‐resolution methods for DNA imaging are available, such as super‐resolution microscopy, AFM, and TEM, none are easy to implement. For example, super‐resolution microscopy demands considerable time and storage capacity for data acquisition. Another crucial requirement for DNA imaging is the compatibility with microfluidic devices and chemically functionalized surfaces. For example, DNA images obtained using fluorescence microscopy often display well‐aligned and parallel elongations.^[^ ^10^ ^]^ This feature is challenging to achieve with TEM, as DNA typically appears randomly arranged due to alignment difficulties on a carbon film‐coated metal grid.

SEM offers a potential solution for obtaining both high‐resolution and well‐aligned DNA images while maintaining simplicity and accessibility. First, SEM inherently provides nanometer‐scale resolution. Second, it offers greater flexibility in accommodating chemically functionalized surfaces on which microfluidic devices can guide and align DNA molecules. However, it is challenging to observe the 2‐nm width of DNA in SEM due to the resolution. Methods such as nanoparticle attachment and nanowire growth have been employed to enhance the visibility of DNA molecules under SEM.^[^ ^11^ ^]^ However, these methods using SEM are primarily used to visualize artificially constructed DNA nanostructures^[^ ^12^ ^]^ and have not been applied to genomic DNA.

Given these challenges, we have developed a new approach for DNA imaging using SEM. With this approach, we have achieved the visualization of DNA obtained from biological samples in SEM for the first time, to the best of our knowledge. Our method combines DNA‐binding proteins with synthetic polymers that serve as electro‐stains. This combination increased the width of the DNA from 2 to 15 nm, which is suitable for SEM imaging. Unlike most electro‐stains, our reagent is metal‐free, allowing DNA to appear as a continuous dark line under SEM. Using this approach, we successfully obtained high‐resolution SEM images of various DNA forms, including linear, circular, double‐stranded, single‐stranded DNA, and RNA. This approach was also employed to visualize genomic and mitochondrial DNA. Moreover, we attached quantum dots (QD) as sequence‐specific bright labels to the dark DNA line. Consequently, our SEM DNA imaging strategy was proven to be both powerful and readily implementable, effectively combining the convenience of fluorescence microscopy with the high‐resolution characteristics of TEM.

## 2 Results and Discussion

### 2.1 SEM Imaging of DNA with Novel Electro‐Stain

**Figure 1:**
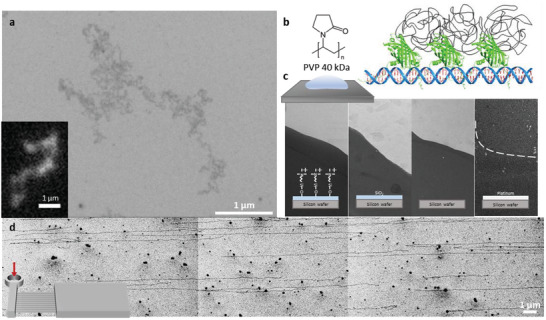
SEM DNA Imaging a) A typical SEM image of bacteriophage λ DNA (48.5 kb), compared with its fluorescence counterpart (inset). b) Schematic illustration of DNA, DNA‐binding proteins (mNG‐HMG: mNeonGreen high mobility group, 31.79 kDa), and synthetic polymers (PVP: polyvinylpyrrolidone, 40 kDa). c) SEM visualization of a PVP droplet on various functionalized silicon wafer surfaces, presented in order from left to right: a positively charged silicon wafer; a SiO_2_ layered silicon wafer; a bare silicon wafer; and a platinum‐coated silicon wafer. The dashed line in the platinum‐coated wafer image delineates the PVP droplet's border. d) SEM image of DNA elongated in parallel and immobilized on a positively charged surface, with an inset showing the microfluidic device. The scale bar represents 1 µm.

**Figure** **1**a presents an SEM image of λ DNA (48.5 kb), revealing more intricate conformational details than the corresponding fluorescence image shown in the inset. Our electro‐stain comprises two primary components: a DNA‐binding protein and a synthetic polymer. As illustrated in Figure 1b, the DNA‐binding protein was mNeonGreen‐high mobility group (mNG‐HMG, 31.79 kDa),^[^ ^13^ ^]^ and the synthetic polymer was PVP (polyvinyl pyrrolidone). A natural question arises: why does DNA appear as a dark line when combined with mNG‐HMG and PVP? To answer this question, we conducted a series of experiments as detailed in Figure S1–S10 (Supporting Information): SEM could not visualize DNA molecules alone, DNA with mNG‐HMG, or DNA with PVP (Figures S1–S3, Supporting Information). In contrast, DNA with PVP became visible under SEM when combined with various DNA‐binding proteins, such as histone‐like nucleoid‐structuring protein (H‐NS, 16 kDa), truncated TALE‐emGFP (63 kDa),^[^ ^14^ ^]^ Cro‐mNeonGreen (37 kDa),^[^ ^15^ ^]^ and H‐NS‐mScarlet (45 kDa)^[^ ^16^ ^]^ (Figures S4–S7, Supporting Information). These DNA‐binding proteins may serve as docking sites for the PVP.^[^ ^17^ ^]^ We also tested various polymers. Among them, poly(2‐ethyl‐2‐oxazoline) and polyaniline exhibited SEM images of DNA (Figures S8 and S9, Supporting Information). In addition, polyaniline alone can bind DNA without the aid of proteins,^[^ ^18^ ^]^ but the resulting image quality is not as good as those of PVP and poly(2‐ethyl‐2‐oxazoline) (Figure S10, Supporting Information), where large aggregates with DNA branches were observed (Figure S10, Supporting Information). Hence, our conclusion is that effective electro‐staining of DNA molecules under SEM necessitates a combination of a DNA‐binding protein and a synthetic polymer capable of binding to the protein.

Subsequently, we examined the surface properties. We used a positively charged Si wafer as the primary platform for immobilizing DNA molecules. Glass surfaces are commonly employed for fluorescence imaging, but they are not suitable for SEM because they induce electron‐charging effects. A typical mitigation strategy involves coating a sample with a thin conductive layer, such as gold or platinum. This method, however, proves unsuitable for SEM imaging of 2‐nm‐thick DNA molecules, as they become obscured–akin to being “buried in snow.” Instead, we utilized a silicon wafer with a 30‐nm oxide layer (SiO_2_). The semiconductive properties of silicon wafers effectively mitigate charging effects,^[^ ^19^ ^]^ and the silicon oxide layer can be derivatized using quaternary ammonium siloxane to immobilize the DNA backbone.^[^ ^10^ ^]^ To explore the influence of surface attributes, we applied a droplet of PVP solution to four different surfaces. After drying, the edges of the droplets were captured using SEM. Figure 1c illustrates four cases: quaternary‐ammonium‐siloxane‐derivatized SiO_2_, bare SiO_2_, bare Si, and platinum‐coated Si wafers. SEM images of the PVP droplet edge revealed a clear delineation between the darker PVP section and the luminous neighboring silicon wafer territory. Notably, this delineation decreased substantially on the platinum‐coated silicon wafer, where the edge was faintly discernible, as indicated by the dashed line. To clarify this, a silicon wafer was first coated with platinum by sputtering and then a droplet of PVP was placed on the wafer. These findings suggest that PVP droplets manifest as dark entities owing to their hindrance to the emission of secondary electrons from semiconductive surfaces. Conversely, a fully conductive surface, such as a platinum‐coated one, emits a surge of secondary electrons that can traverse the surface PVP layer, leading to diminished contrast. Therefore, a semiconductive Si wafer is a pivotal component in SEM DNA imaging.

Furthermore, the surface of the silicon wafer offers the advantage of easily mounting a PDMS microfluidic device that guides the DNA solution through the microchannels.^[^ ^20^ ^]^ Figure 1d shows DNA molecules that were elongated in parallel. These were steered by capillary force within a microfluidic channel and subsequently immobilized on the positively charged surface of the silicon oxide layer. This process is essential for DNA elongation. However, implementing this method is challenging in carbon‐film‐based TEM imaging of DNA molecules. As a result, most TEM DNA images documented to date exhibit random arrangements,^[^ ^21^ ^]^ similar to those observed in Figure 1a.

### 2.2 15‐nm Thickness of DNA Backbone Under SEM

**Figure 2:**
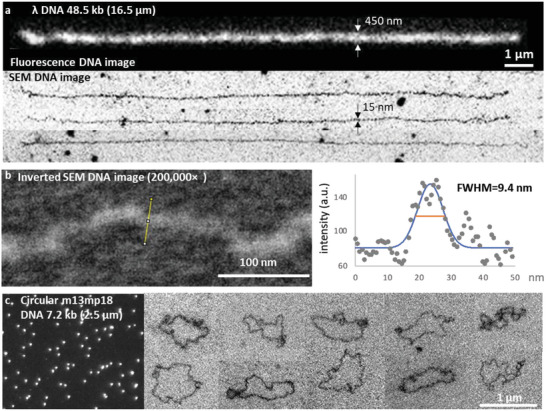
SEM DNA image resolution of 15 nm. (a) A comparison of the resolution of fluorescence and SEM DNA images using DNA stained with mNG‐HMG and PVP. b) The minimal full width at half maximum (FWHM) of 9.4 nm was measured using the intensity profile depicted along the yellow line. c) Various shapes of SEM images of circular DNA (7.2 kb) with their corresponding fluorescence images of the dots.

**Figure** **2** shows the resolution of SEM images of DNA, comparing them with fluorescence images. While the lengths of the λ DNA molecules remain consistent, a significant difference was observed in their respective thicknesses. SEM imaging showed a DNA thickness of 15 nm, contrasting sharply with the 450 nm thickness observed in a fluorescence image. For precision, we determined the full width at half maxima (FWHM) of the intensity profile (*see* SI ImageJ Macro “doFWHM” downloaded from GitHub). The FWHM of the SEM DNA images averages 15.0 ± 4.0 nm, ranging from 9.4 to 22.8 nm, while the FWHM of fluorescence DNA images averages 239 ± 30 nm. The graph on the right illustrates an instance where the FWHM is 9.4 nm, corresponding to the yellow line in the SEM DNA image on the left. This thickness may involve the sizes of the three pivotal components: DNA, proteins, and polymers (Figure 1b). The DNA is 2‐nm wide. The dimension of mNG‐HMG depends on the fluorescent protein β‐barrel which is 2.4 nm in diameter and 4.2 nm in height. The linear polymer PVP weighs 40 kDa and has an *R*_g_ of 3.8 nm based on a reference value.^[^ ^22^ ^]^ Thus, the external diameter of the PVP was ≈9.8 nm, as deduced from $d_{out}=\;2\sqrt{5/3}R_{g}$, assuming a spherical shape. This figure closely aligns with the 9.4‐nm FWHM in Figure 2b. The aggregation of proteins and polymers encircling the DNA likely contributes to the observed average thickness of 15.0  ± 4.0 nm. Notably, this high‐resolution captures the circular or supercoiled DNA configuration, which merely appears as small dots in the fluorescence images (Figure 2c).

Our SEM‐based DNA imaging method offers a high‐resolution approach to reveal detailed structures. However, it is crucial to compare this method with other currently available high‐resolution DNA imaging methods, such as cryo‐EM, TEM, AFM, fluorescence microscopy, and super‐resolution microscopy. Cryo‐EM boasts the highest reported resolution, achieving 2‐nm diameter visualization without any staining.^[^ ^6a^ ^]^ Typical TEM studies have reported DNA resolutions ranging from 4 to 6 nm after uranyl acetate staining.^[^ ^23^ ^]^ The thickness of the metal casting is typically ≈8 nm for high‐contrast TEM images.^[^ ^24^ ^]^ AFM studies have demonstrated that the diameter of DNA ranges between 5 and 10 nm, although they have also revealed a sub‐nanometer helical groove pattern within the DNA backbone.^[^ ^25^ ^]^ Furthermore, several studies utilizing super‐resolution microscopy methods have been reported, with the finest resolution for DNA imaging being 45 nm using STED (Stimulated Emission Depletion),^[^ ^8a^ ^]^ 14 nm using BALM (Binding Activated Localization Microscopy),^[^ ^8b^ ^]^ 67 nm using STORM (Stochastic Optical Reconstruction Microscopy),^[^ ^26^ ^]^ and 20 nm using Vortex PSF (Point Spread Function).^[^ ^27^ ^]^ Given these comparisons, the 15‐nm resolution obtained in our SEM method presents an attractive option for various genomic applications.

### 2.3 SEM Images of Various Types of DNA Molecules Extracted from HeLa Cells

**Figure 3:**
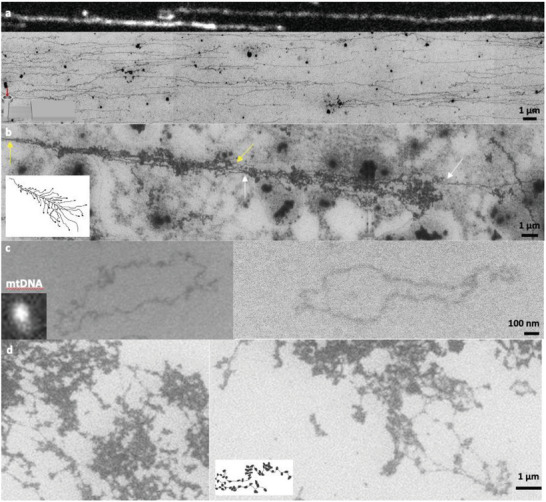
SEM images of various DNA types extracted from HeLa cells. a) Comparison of fluorescence and SEM images of genomic DNA obtained from HeLa cells. A microfluidic device (inset) and positively charged surface facilitated the elongation of DNA in parallel. b) Christmas‐tree‐like DNA images potentially indicative of transcription along the DNA backbone. c) Contrasting fluorescence microscopy and SEM depictions of circular mitochondrial DNA (16.6 kb). d) Chromatin structures to represent chromatin fibers and their aggregations.

The ultimate objective of SEM DNA imaging is to visualize DNA molecules derived from human cells. To this end, we prepared genomic DNA samples from HeLa cells. **Figure** **3**a displays SEM images of elongated and immobilized genomic DNA, along with a comparison to fluorescence images. The fluorescence image depicts a thick, tangled DNA structure, posing challenges in elucidating its conformation. Notably, significant variation in fluorescence intensity was observed in the DNA image. In contrast, the SEM image clearly reveals the DNA and its spatial distribution. Multiple closely located DNA molecules are discernible in the SEM image, a feature not apparent in the fluorescence image.

Another notable observation is the “Christmas‐tree‐like” structure as shown in Figure 3b. This structure is believed to represent DNA transcription, as documented in earlier studies^[^ ^4^, ^28^ ^]^ which may not be discernible in fluorescence images owing to diffraction limits. Such TEM image structures have been reported in amphibian oocytes,^[^ ^4a^ ^]^ insects,^[^ ^28b^ ^]^ yeast,^[^ ^28c^ ^]^ and rats.^[^ ^28a^ ^]^ To the best of our knowledge, no TEM images depicting this structure have been reported in human cells or cell lines. Figure 3b shows the first EM image of DNA transcription from a human cell. In Figure 3b, the yellow and white arrows indicate the DNA backbone, whereas the progressively denser brush‐like lines represent transcribed RNA molecules. A pertinent question is how RNA is stained with HMG, given that HMG binds to DNA molecules. Given the six positively charged amino acid residues (3K and 3R) in the TPKRPRGRPKK sequence, HMG was expected to demonstrate an affinity for the negatively charged backbones of RNA. One must note that HMG binds to RNA with affinity.^[^ ^29^ ^]^ Furthermore, considering the cellular origin of the sample, RNA molecules are likely associated with RNA‐binding proteins, to which PVP may bind. However, SEM imaging for RNA molecules tends to be inefficient because single‐stranded RNA molecules are usually small and prone to aggregation, complicating microscopic observation.

Figure 3c provides a comparative view of the fluorescence and SEM images of the mitochondrial DNA extracted from HeLa cells. Although this DNA is 16.6 kb in length, it appears as a dot in the fluorescence image. In contrast, the SEM image reveals an intricate conformation of the circular DNA, reminiscent of the circular DNA shown in Figure 2c.^[^ ^30^ ^]^

In addition, we aimed to demonstrate chromatin structures through our approach, as depicted in Figure 3d. These structures were revealed by lysing cells that were arrested in metaphase with a surfactant (Triton X‐100), resulting in the partially unraveled chromosomal packing.

### 2.4 Sequence‐Specific Quantum Dot Labels

**Figure 4:**
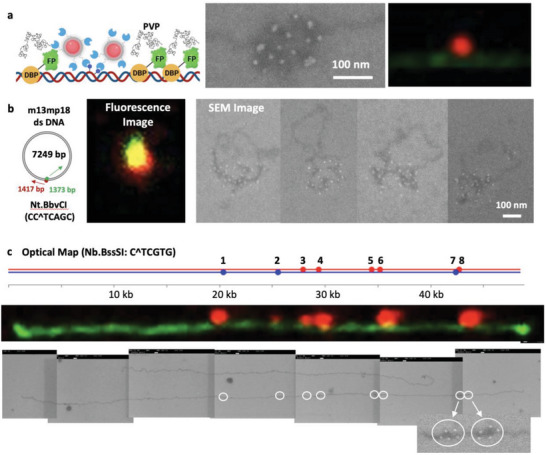
SEM image of DNA labeled with quantum dots. a) Quantum dot‐labeled DNA in SEM and fluorescence images. The schematic represents DNA labeled with streptavidin (blue)‐coated quantum dots (pink) and stained with mNG‐HMG (FP‐DBP) and PVP (black coil). b) Quantum dot‐labeled circular DNA (7.2 kb) in fluorescence and SEM images. Arrows in the schematic indicate polymerase directions during nick‐translation. c) Optical mapping of λ DNA using Nb.BssSI (C^TCGTG) and streptavidin‐coated quantum dot labels, with eight sites depicted in the in silico map, six labels in the fluorescence optical map, and eight labels in the SEM optical map. The magnified SEM image represents the 7^th^ and 8^th^ sites with a distance of 321 bp (109 nm).

In SEM images, DNA appears as a dark line, providing a contrasting platform for labeling nanoparticles, which appear as bright objects. Consequently, we utilized this contrast by attaching quantum dots to DNA. **Figure** **4** presents the SEM images of DNA with attached quantum dots. To achieve this, one strand of DNA is digested by sequence‐specific nickases such as Nb.BbvCI (CC^TCAGC). Subsequently, DNA polymerase incorporates biotin‐labeled dUTP into the DNA via nick translation.^[^ ^17^ ^]^ The streptavidin‐coated quantum dot binds bound to biotins on the DNA. Figure 4a displays multiple quantum dots in the SEM image, while these quantum dots appeared as single dots in the fluorescence image. Figure 4b presents an example of circular DNA (7.2 kb) labeled with quantum dots. The fluorescence image reveals a bright yellow spot resulting from the merging of the green fluorescence and red quantum dot signals. In contrast, individual SEM images clearly demonstrate 20–30 quantum dot labels on the circular DNA backbone.

Next, we explored the potential of SEM for nickase‐based optical DNA mapping. We hypothesized that SEM‐based optical DNA mapping would surpass the resolution offered by fluorescence‐based optical DNA mapping.^[^ ^31^ ^]^ As a model system, we selected λ DNA and Nb. BssSI, which cleaves at the C^TCGTG site, generates eight labeling sites (Figure 4c). However, due to the short distances between neighboring sites, it was challenging to observe all eight in the fluorescence image; the 5^th^ and 6^th^ sites are 789 bp apart (789 bp × 0.34 nm/bp = 268 nm), and the 7^th^ and 8^th^ sites are 321 bp apart (321 bp × 0.34 nm/bp = 109 nm). As a result, only six red labels, as shown in Figure 4c, are distinct in the fluorescence image. In contrast, the SEM optical maps successfully displayed all eight sites. As demonstrated by the magnified images of the 7^th^ and 8^th^ sites, five QDs were labeled at each site. Each site, comprising multiple nanoparticles, confirmed the precise location of sequence‐specific nick translation.

The results presented in Figure 4c hold promise for SEM‐based optical DNA mapping. Over the decades, various methods have been developed to extract sequence information from DNA images, including in situ hybridization,^[^ ^32^ ^]^ enzymatic digestion by restriction enzymes,^[^ ^33^ ^]^ fluorochrome labeling via nick‐translation,^[^ ^31a^ ^]^ and methyl transferase.^[^ ^34^ ^]^ Additionally, methods such as DNA melting maps^[^ ^35^ ^]^ and hybridization^[^ ^36^ ^]^ have been developed using TEM to reveal sequence information from TEM DNA images. However, TEM encounters limitations in analyzing large DNA molecules due to sampling issues. Consequently, some fluorescence‐based technologies have been commercialized to provide genome analysis platforms.^[^ ^31b^ ^]^ In comparison, SEM DNA mapping offers several advantages. First, it boasts higher resolution compared to fluorescence‐based optical DNA mapping. Figure 4c demonstrates the example of high‐resolution DNA optical mapping using SEM. Due to the resolution limit of fluorescence microscopy, sites 5,6 and sites 7,8 appear overlapped, resulting in the observation of six labels instead of eight. However, in SEM optical DNA mapping, all eight sites are individually observed, enabling precise DNA optical mapping, and consequently providing accurate sequence information of DNA. Second, SEM optical DNA mapping allows for multiplexing through the utilization of various nanoparticles,^[^ ^11^, ^12^ ^]^ thus overcoming issues related to fluorescence spectrum overlap. Third, nanoparticle labels exhibit exceptional stability without facing issues such as fluorochrome inactivation, like photo‐bleaching. Fourth, SEM optical DNA mapping does not encounter issues related to DNA sample size, unlike TEM DNA imaging. Consequently, SEM optical DNA mapping holds significant promise as a genome analysis platform, potentially replacing the current optical mapping platform that utilizes fluorescence microscopy.

## 3 Conclusion

In this study, we present a novel approach to SEM imaging that helps visualize various DNA structures. Our method employs DNA‐binding proteins and synthetic polymers as staining reagents to render DNA visible as a dark line with an average thickness of 15.0 ± 4.0 nm. This novel staining approach for SEM offers a valuable tool for various genomic applications, including genomic DNA visualization and SEM‐based optical DNA mapping. It offers a simpler and more user‐friendly alternative to other imaging methods, and its compatibility with microfluidic devices and functionalized surfaces makes it a significant tool for genomics researchers. We believe that SEM‐based DNA imaging opens new possibilities for high‐resolution visualization of DNA structures and related biomolecules, as well as high‐resolution SEM‐based optical DNA mapping. Given the versatility and widespread popularity of SEM among analytical instruments, SEM‐based DNA imaging will be  a well‐positioned standard tool for genomic analysis, offering insight into numerous biochemical reactions involving large DNA molecules.

## 4 Experimental Section

### Chemicals

Polyvinylpyrrolidone (PVP), poly(2‐ethyl‐2‐oxazoline), and polyaniline were purchased from Sigma‐Aldrich (St. Louis, MO, USA). Qdot 585 streptavidin conjugate was purchased from Thermo Fisher Scientific (Waltham, MA, USA). Oxidized silicon wafers were purchased from the Wafer Market (Yong‐In, Korea). *N*‐Trimethoxysilylpropyl‐*N, N, N*‐trimethylammonium chloride was purchased from Gelest (Morrisville, PA, USA). Bacteriophage λ DNA (48.5 kb), m13mp18 double‐stranded DNA (7.2 kb), Nb.BssSI, and DNA polymerase I were purchased from New England Biolabs (Ipswich, MA, USA). T4 GT7 DNA was purchased from Nippon Gene (Toyama, Japan). Biotin‐16‐dUTP was purchased from Jena Biosciences (Tokyo, Japan). AMPure XP beads were purchased from Beckman Coulter.

### DNA‐Binding Protein (mNeonGreen‐HMG)

The DNA‐binding motif of the high‐mobility group (HMG: TPKRPRGRPKK), as previously described,^[^ ^13^, ^37^ ^]^ was fused to the C‐terminal region of mNeonGreen (mNG), provided by Dorus Gadella (Addgene plasmid # 98 882, Addgene, Watertown, MA, USA), using polymerase chain reaction (PCR). mNG‐HMG was prepared using overlap extension PCR. The amplified product was digested using restriction enzymes NdeI (CA^TATG) and BamHI (G^GATCC). It was then inserted into the pET15b vector. The amino acid sequence of mNG‐HMG is GSSHHHHHHSSGLVPRGSHMMLPGCMLMVSKGEEDNMASLPATHELHIFGSINGVDFDMVGQGTGNPNDGYEELNLKSTKGDLQFSPWILVPHIGYGFHQYLPYPDGMSPFQAAMVDGSGYQVHRTMQFEDGASLTVNYRYTYEGSHIKGEAQVKGTGFPADGPVMTNSLTAADWCRSKKTYPNDKTIISTFKWSYTTGNGKRYRSTARTTYTFAKPMAANYLKNQPMYVFRKTELKHSKTELNFKEWQKAFTDVMGMDELYKLETPKRPRGRPKKMLGSGC (283 residues, 31.79 kDa).

The constructed mNG‐HMG plasmid was transformed into *E. coli* BL21 (DE3). The BL21 cells harboring the mNG‐HMG plasmid were then incubated in a Luria Broth (LB) medium supplemented with ampicillin at 37 °C for 16 h overnight. Next, 1 mL of the incubated culture was transferred to 100 mL of an LB medium containing ampicillin and incubated at 37 °C until the optical density at 600 nm (OD_600_) reached 0.4–0.6. For induction, 1 mM IPTG was added, followed by incubation for 16 h overnight at 20–25 °C with shaking at 200 rpm. After ultrasonication for 15 min and centrifugation for 10 min at 10 000 rpm (12 298 × *g*), mNG‐HMG was harvested by affinity chromatography using Ni‐NTA agarose resin. mNG‐HMG was eluted with a buffer containing 50 mm Na_2_HPO_4_, 300 mm NaCl, and 250 mm imidazole at pH 8.0. The purified proteins were buffer‐exchanged with phosphate‐buffered saline (PBS; 137 mm NaCl, 2.7 mm KCl, 10 mm Na_2_HPO_4_, and 1.8 mm KH_2_PO_4_, pH 7.4) and stored at −20 °C with the addition of glycerol. Details of the other DNA‐binding proteins are available in Supplementary Information (SI).

### Preparation of Positively Charged Silicon Wafer Surface

Silicon wafers with a 30‐nm SiO_2_ layer were purchased from Wafer Market (Yong‐In, Korea). The method established for glass coverslips^[^ ^10^ ^]^ was adapted to treat the SiO_2_ layer and create positively charged surfaces. The wafers were incubated in a 250‐mL solution containing *N*‐trimethoxysilylpropyl‐*N, N, N*‐trimethylammonium chloride (1.1 mm) at 65 °C with agitation at 100 rpm for 16 h. They were then rinsed three times with 99.9% ethanol and stored in 99.9% ethanol.

### SEM Imaging of DNA Molecules

The DNA molecules were mixed with mNG‐HMG and incubated for 10 min. After adjusting the concentration with 1× TE buffer (Tris 10 mm and EDTA 1 mm, pH 8.0), an equal volume of 5% PVP (40 KDa) solution was added, followed by an additional 5‐min incubation. Briefly, 1 µL of the solution was loaded onto a positively charged silicon wafer. It was important to ensure that the solution was completely dried prior to the SEM observation. Using SEM, the DNA was observed at magnifications ranging from 5000 to 50 000×. For image processing, ImageJ 1.53a (National Institute of Health, Bethesda, MD, USA) was used in conjunction with the “doFWHM” macro (refer to SI) to measure the resolution of the DNA images. For aligning DNA in parallel, a polydimethylsiloxane (PDMS) microchannel was utilized as previously described,^[^ ^20^ ^]^ with device dimensions of 100 µm in width and 2.4 µm in height. Before detaching from the PDMS microchannel, the solution was dried for 50 min. Control fluorescence images presented in the figures were acquired using a previously described method.^[^ ^38^ ^]^

### DNA Preparation from HeLa Cell: HeLa Cell Preparation

HeLa cells were cultured in a Dulbecco's Modified Eagle Medium (DMEM) (high glucose, pyruvate) supplemented with a final concentration of 10% fetal bovine serum and 1× antibiotic‐antimycotic (Gibco, Waltham, MA, USA). When the HeLa cells reached ≈80% confluence in the cell culture dish, the medium was carefully aspirated using a Pasteur pipette. Subsequently, 5 mL of 1× PBS (137 mm NaCl, 2.7 mm KCl, 10 mm Na_2_HPO_4_, and 1.8 mm KH_2_PO_4_, pH 7.4) was gently added to the side of the dish and then aspirated. Next, 2 mL of trypsin‐EDTA buffer was added, and the dish was shaken by hand to ensure coverage of the bottom. The cells were incubated for 2 min. Next, 8 mL of medium was added, and the total solution (10 mL) was transferred into a 15‐mL conical tube and centrifuged at 500 × *g* for 10 min. HeLa cells were resuspended in 1 mL of 1× PBS and transferred to an Eppendorf tube. After washing twice with a 1× PBS buffer, the cells were finally resuspended in 500 µL of 1× PBS buffer.

### DNA Preparation from HeLa Cell: Genomic DNA Preparation

Genomic DNA was extracted using agarose gel plugs. The cell solution was mixed with a 2% 1× TE Low Gelling Temperature (LGT) Agarose solution to achieve a final LGT concentration of 0.7% and then incubated for 30 min at 4 °C. The DNA plug was washed with 1× TE buffer for 30 min, and this step was repeated four times. Proteinase K was added to a final concentration of 2 mg mL^−1^, and the mixture was incubated for 4 h at 42 °C. An equal amount of proteinase K was added again, followed by 16 h overnight incubation at 42 °C. The DNA plug was then washed for 30 min with 1× TE buffer, and this step was repeated four times. Finally, 380 µL of 1× TE buffer was added, and the DNA plug was melted by incubating it at 65 °C for 20 min.

### DNA Preparation from HeLa Cell: Mitochondrial DNA Preparation

Mitochondrial DNA was prepared using a QIAprep Spin Miniprep Kit (Qiagen, Hilden, Germany). The cell suspension was centrifuged at 1000 × *g* for 10 min. After discarding the supernatant, the cells were resuspended in 300 µL of P1 buffer. Next, 300 µL of P2 buffer was added, followed by the addition of 420 µL of N3 buffer. The tube was then centrifuged at 12000 × *g* for 10 min. The resulting supernatant was transferred to a QIAprep spin column (Qiagen) and centrifuged at 12000 × *g* for 1 min. The plasmid was eluted with 100 µL of elution buffer. After the volume of the eluted solution was accurately measured, 0.4 times the volume of AMPure XP beads were added. The solution was mixed well for 5 min to ensure proper binding of DNA to the beads. The beads were allowed to bind to the magnet for 1 min using a magnetic separation rack. The solution was then slowly removed from the opposite side of the magnet, and 200 µL of 80% ethanol was added. The rack was gently inverted 5 times and the ethanol was removed. This process was repeated twice. Any remaining ethanol was evaporated by leaving the tube open for ≈5 min at room temperature. The tube was removed from the rack, and 25 µL of 0.1 × TE buffer was added to the beads and mixed well. After incubating at room temperature for 10 min, the magnetic separation rack was used again to allow the beads to bind to the magnet for 1 min. The solution was then transferred to another tube, thereby isolating mitochondrial DNA.

### Quantum Dot Labeled DNA Molecules

Quantum dot labeling was performed by nick translation after nickase (Nb.BbvCI) treatment following a previously described method.^[^ ^17^ ^]^ 500 ng of M13mp18 double‐stranded DNA was incubated with 20 units of Nb.BbvCI and 10 units of DNA polymerase I in NEB buffer 2 at 16 °C for 2 h. The mixture for heavy labeling included biotin‐labeled dUTP (1.1 µM), TTP (1.1 µM), dATP (11 µM), dCTP (11 µM), and dGTP (11 µM). However, for multiple labeling, the mixture contained 1.1 µM of each nucleotide. After the nick translation, the DNA solution was diluted to a final concentration of 10 ng µL^−1^. This solution was mixed with 30 nm of streptavidin‐conjugated quantum dot (Qdot 585) and incubated at 20–25 °C for 10 min. To separate the DNA from unbound Qdot 585, a 25‐nm mixed cellulose esters membrane was placed on top of 100 µL of 1× TE buffer. The DNA solution was added to the membrane and incubated for 30 min. The DNA solution in the membrane was then carefully retrieved. A total of 500 ng of bacteriophage λ DNA was incubated with 20 units of Nb.BssSI and 10 units of DNA polymerase I in NEB buffer 2 at 16 °C for 2 h. The mixture for labeling included biotin‐labeled dUTP (1.1 µM), TTP (1.1 µM), dATP (1.1 µM), dCTP (1.1 µM), and dGTP (1.1 µM) as previously described above.

## Conflict of Interest

The authors declare no conflict of interest.

## Supporting information

Supporting Information

## Data Availability

The data that support the findings of this study are available from the corresponding author upon reasonable request.
