# Supplementary material for: Scanning Electron Microscopy Imaging of Large DNA Molecules Using a Metal‐Free Electro‐Stain Composed of DNA‐Binding Proteins and Synthetic Polymers
Source: Adv Sci (Weinh). 2024 May 5;11(28):2309702. doi: 10.1002/advs.202309702 (PMC11267313; doi:10.1002/advs.202309702)
Supplement: Supplementary file 1 — Supporting Information [file ADVS-11-2309702-s001.docx]

**SEM Imaging of Large DNA Molecules Using a Metal-Free Electro-Stain Composed of DNA-Binding Proteins and Synthetic Polymers**

Changyoung Noh^1^, Yoonjung Kang^1^, Sujung Heo^1^, Taesoo Kim^1^, Hayeon Kim^1^, Junhyuk Chang^2^, Priyannth Ramasami Sundharbaabu^2^, Sanghee Shim^3^, Kwang-il Lim^4^, Jung Heon Lee^2,5^*, Kyubong Jo^1^*

^1^Department of Chemistry, Sogang University; Seoul 04107, Korea

^2^School of Advanced Materials Science and Engineering, Sungkyunkwan University (SKKU); Suwon 16419, Korea

^3^Department of Chemistry, Korea University; Seoul 02841, Korea

^4^Department of Chemical and Biological Engineering, Sookmyung Women's University; 04312, Seoul, Korea

^5^Department of MetaBioHealth, Sungkyunkwan University (SKKU); Suwon 16419, Korea.

*Corresponding author. Email: jokyubong@sogang.ac.kr, jhlee7@skku.edu

**Table of content**

Materials and Methods

- DNA-binding protein

- ImageJ macro for DNA thickness measurement

Figs. S1 to S10

References

**Materials and Methods**

**DNA-binding protein**

**1) Trunacted TALE (tTALE)-emGFP**

The tTALE-emGFP plasmid was prepared as previously described [1]. Briefly, the tTALE-emGFP plasmid was generated through an extension polymerase chain reaction that connects the emGFP fluorescent protein to the C-terminal region of the DNA-binding protein TALE. The bridging amino acid sequence was GGSGG. The constructed tTALE-EmGFP plasmid was transformed into *E. coli* BL21 (DE3) strains using a standard cloning procedure. The amino acid sequence for tTALE-EmGFP is described below.

MGSSHHHHHHSSGLVPRGSHMDLRTLGYSQQQQEKIKPKVRSTVAQHHEALVGHGFTHAHIVALSQHPAALGTVAVKYQDMIAALPEATHEAIVGVGKQWSGARALEALLTVAGELRGPPLQLDTGQLLKIAKRGGVTAVEAVHAWRNALTGAPLNLTPAQVVAIASNNGGKQALETVQRLLPVLCQDHGLTPAQVVAIASNGGGKQALETVQRLLPVLCQAHGLTPDQVVAIASHDGGKQALETVQRLLPVLCQDHGLTPAQVVAIASNGGGKQALETVQRLLPVLCQAHGLTPDQVVAIASNNGGKQALETVQRLLPVLCQAHGLTPAQVVAIASNGGGKQALETVQRLLPVLCQDHGGGSGGMVSKGEELFTGVVPILVELDGDVNGHKFSVSGEGEGDATYGKLTLKFICTTGKLPVPWPTLVTTFAYGLQCFARYPDHMKQHDFFKSAMPEGYVQERTISFKDDGNYKTRAEVKFEGDTLVNRIELKGTDFKEDGNILGHKLEYNYNSHNVYITADKQKNGIKANFKIRHNIEDGSVQLADHYQQNTPIGDGPVLLPDNHYLSTQSALSKDPNEKRDHMVLLEFVTAAGITLGMDELYK (64.7 kDa)

**2) H-NS**

The H-NS plasmid was constructed by amplifying H-NS gene from H-NS-mCherry [2]. Plasmid pET15b was digested with the restriction enzymes *Nde*I (CA^TATG) and *BamH*I (G^GATCC). Then, the H-NS gene was inserted and ligated into the plasmid using the AccuRapid Cloning Kit, creating the H-NS plasmid. The constructed H-NS plasmid was transformed into *E. coli* BL21 (DE3) strains using a standard cloning procedure. The amino acid sequence for H-NS is described below. MGSSHHHHHHSSGLVPRGSHMMSEALKILNNIRTLRAQARECTLETLEEMLEKLEVVVNERREEESAAAAEVEERTRKLQQYREMLIADGIDPNELLNSLAAVKSGTKAKRAQRPAKYSYVDENGETKTWTGQGRTPAVIKKAMDEQGKSLDDFLIKQGSGC (18.10 kDa)

**3) H-NS-mScarlet**

The H-NS-mScarlet plasmid was prepared as previously described [3]. Briefly, H-NS plasmid was digested with *BamH*I (G^GATCC). Then the amplified mScarlet gene was also inserted and ligated into the H-NS plasmid using the AccuRapid Cloning Kit. The constructed H-NS-mScarlet plasmid was transformed into *E. coli* BL21 (DE3) strains using a standard cloning procedure. The amino acid sequence for H-NS-mScarlet is described below.

MGSSHHHHHHSSGLVPRGSHMMSEALKILNNIRTLRAQARECTLETLEEMLEKLEVVVNERREEESAAAAEVEERTRKLQQYREMLIADGIDPNELLNSLAAVKSGTKAKRAQRPAKYSYVDENGETKTWTGQGRTPAVIKKAMDEQGKSLDDFLIKQEFMVSKGEAVIKEFMRFKVHMEGSMNGHEFEIEGEGEGRPYEGTQTAKLKVTKGGPLPFSWDILSPQFMYGSRAFTKHPADIPDYYKQSFPEGFKWERVMNFEDGGAVTVTQDTSLEDGTLIYKVKLRGTNFPPDGPVMQKKTMGWEASTERLYPEDGVLKGDIKMALRLKDGGRYLADFKTTYKAKKPVQMPGAYNVDRKLDITSHNEDYTVVEQYERSEGRHSTGGMDELYKGSGC (44.75 kDa)

**4) Cro-mNeongreen**

The Cro-mNeongreen plasmid was previously described [4]. Briefly, it was created using a cassette vector system to insert the DNA binding protein coding gene, Cro, into the mNeongreen plasmid. The mNeongreen plasmid was generated by inserting the mNeongreen gene between the *Nde*I (CA^TATG) and *Xma*I (C^CCGGG) restriction sites in the pET15b vector. The Cro gene was amplified by PCR using bacteriophage λ DNA as the template. The constructed Cro-mNeongreen plasmid was transformed into *E. coli* BL21 (DE3) strains using a standard cloning procedure. The amino acid sequence for Cro-mNeongreen is described below.

MGSSHHHHHHSSGLVPRGSHMMEQRITLKDYAMRFGQTKTAKDLGVYQSAINKAIHAGRKIFLTINADGSVYAEEVKPFPSNKKTTAGGSGGPGMVSKGEEDNMASLPATHELHIFGSINGVDFDMVGQGTGNPNDGYEELNLKSTKGDLQFSPWILVPHIGYGFHQYLPYPDGMSPFQAAMVDGSGYQVHRTMQFEDGASLTVNYRYTYEGSHIKGEAQVKGTGFPADGPVMTNSLTAADWCRSKKTYPNDKTIISTFKWSYTTGNGKRYRSTARTTYTFAKPMAANYLKNQPMYVFRKTELKHSKTELNFKEWQKAFTDVMGMDELYKGSGC (37.07 kDa)

**ImageJ macro for DNA thickness measurement**

A FWHM macro was downloaded from GitHub to accurately measure the thickness of DNA under SEM. <https://gist.github.com/romainGuiet/42395587d1193536b7a0858f72db4aca>

This macro profiles the intensity of DNA, which is observed as a black line under SEM, and measures the profile's full width at half maximum (FWHM). We utilized FWHM to assess the resolution of DNA molecules under SEM, as it is commonly employed for measuring the resolution of spectrometers. The FWHM macro was employed as an ImageJ plugin, and the macro code is described below.

Macro “FWHM” {

doAxialMeasure = true;

//get some informations about the image

image_Name = getTitle();

getDimensions(image_width, image_height, image_channels, image_slices, image_frames);

getVoxelSize(voxel_width, voxel_height, voxel_depth, voxel_unit);

doFWHM();

if (doAxialMeasure){

// define the length of the line

lineLength = 40;

// to find bright spot , https://imagej.nih.gov/ij/docs/guide/146-29.htmL

prominence = 100 ;

selectImage(image_Name);

run("Reslice [/]...", "output=["+voxel_depth+"]");

run("Find Maxima...", "prominence="+prominence+" output=[Point Selection]");

// get coordinates of the Maxima(s)

getSelectionCoordinates(xpoints, ypoints);

// make a Line using the Coordinates of the First Maxima (the brightest)

// and the line length defined

makeLine(xpoints[0], ypoints[0] - lineLength/2,xpoints[0], ypoints[0] + lineLength/2 );

// do the measure

doFWHM();

}

// companion function to make the math for FWHM

function doFWHM(){

image_Name = getTitle();

getVoxelSize(voxel_width, voxel_height, voxel_depth, voxel_unit);

y = getProfile();

x = Array.getSequence(lengthOf(y));

Fit.doFit("Gaussian", x, y) ;

Fit.plot;

// parameter d of gaussian

sortedParameter = Fit.p(3);

rSquared = Fit.rSquared ;

// http://fr.wikipedia.org/wiki/Largeur_%C3%A0_mi-hauteur

FWHM = (2 * sqrt( 2 * log(2) ) ) * sortedParameter ;

setResult("FWHM ("+voxel_unit+")", nResults, FWHM * voxel_height);

setResult("Label",nResults-1,image_Name);

setResult("rSquared",nResults-1,rSquared);

updateResults();

selectWindow("Results");

}

**Supplementary Figures**


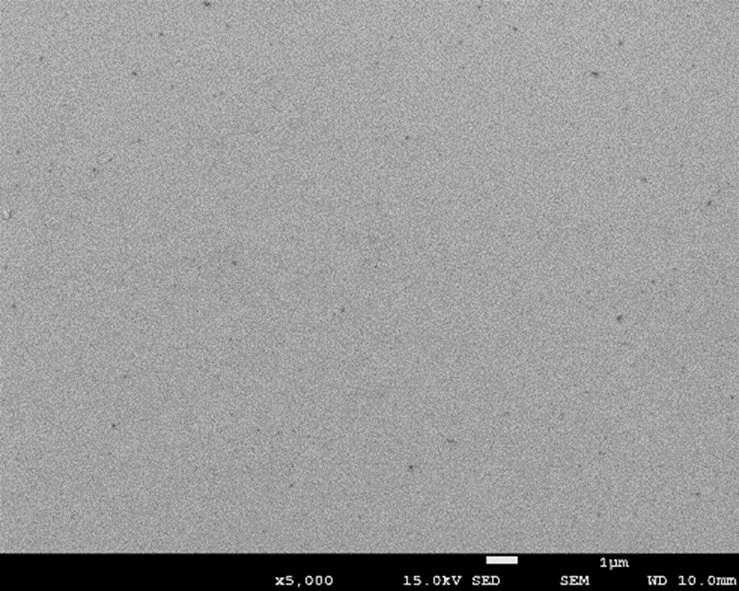


Fig. S1. DNA only under SEM. 25 pg λ DNA (48.5 kb) was elongated and immobilized onto a positively charged silicon wafer in microfluidic device [5]. The surface derivatization was performed with 1.1 mM Q-siloxane (N-trimethoxysilylpropyl-N,N,N-trimethyl ammonium chloride). The SEM image was acquired in SED (secondary electron detector) mode at 15 kV, ×5,000 magnification.


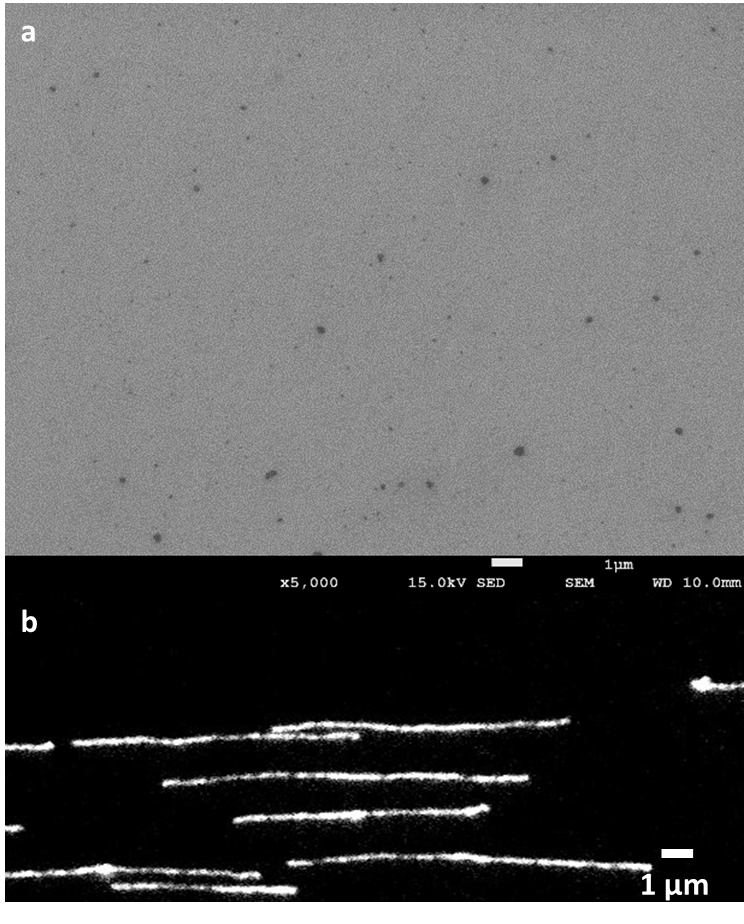


Fig. S2. mNeongreen-HMG stained DNA. a, 25 pg of λ DNA stained with mNeongreen-HMG (31 kDa) at a concentration of 792 nM [1b, 6]. DNA molecules were elongated and immobilized onto a positively charged silicon wafer (coated with 1.1 mM Q-siloxane) inside a microfluidic device. The SEM image was acquired in SED mode at 15 kV, ×5,000 magnification. b, The corresponding FM image.


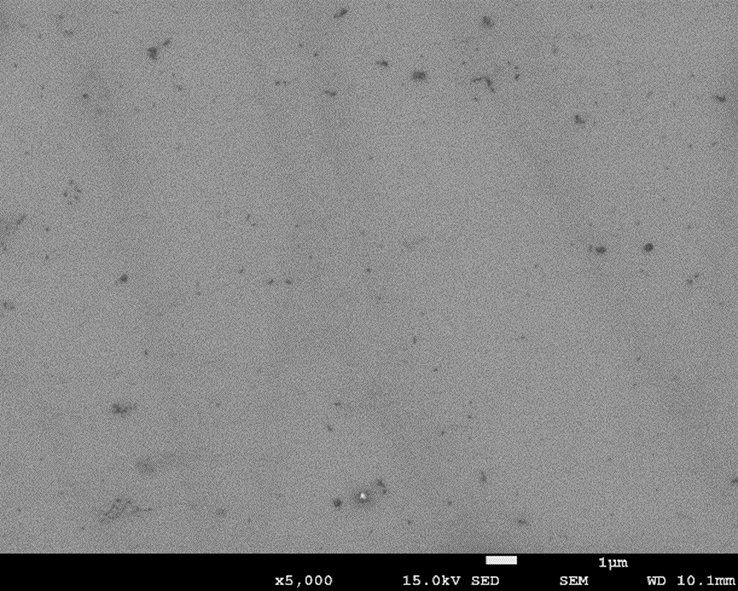


Fig. S3. PVP and DNA mixture under SEM. 25 pg of λ DNA mixed with PVP (polyvinylpyrrolidone; 40 kDa, 5 % in water) solution. DNA molecules were elongated and immobilized onto a positively charged silicon wafer (coated with 1.1 mM Q-siloxane) inside a microfluidic device. The SEM image was acquired in SED mode at 15 kV, ×5,000 magnification.


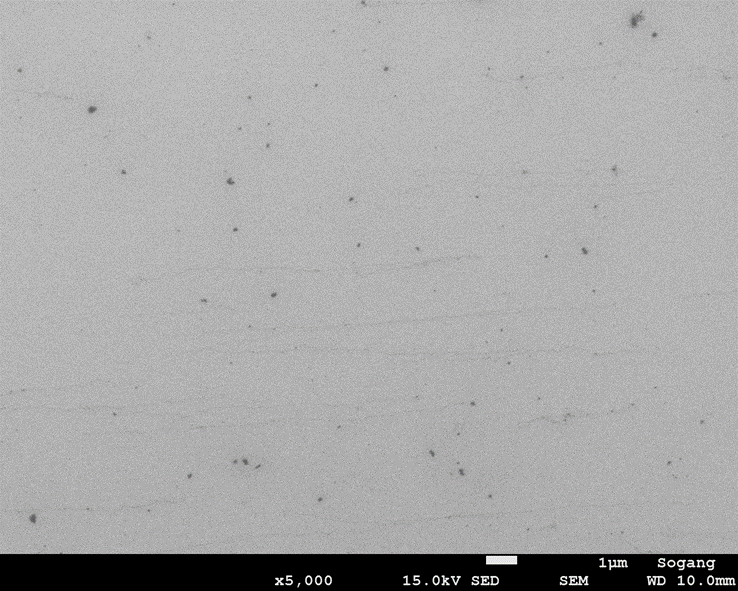


Fig. S4. H-NS and PVP stained DNA. 25pg of λ DNA stained with a DNA binding protein H-NS (18 kDa, 264 nM) and PVP solution (40 kDa, 5% in water). DNA molecules were elongated and immobilized onto a positively charged silicon wafer (coated with 1.1 mM Q-siloxane) inside a microfluidic device. The SEM image was acquired in SED mode at 15 kV, ×5,000 magnification.


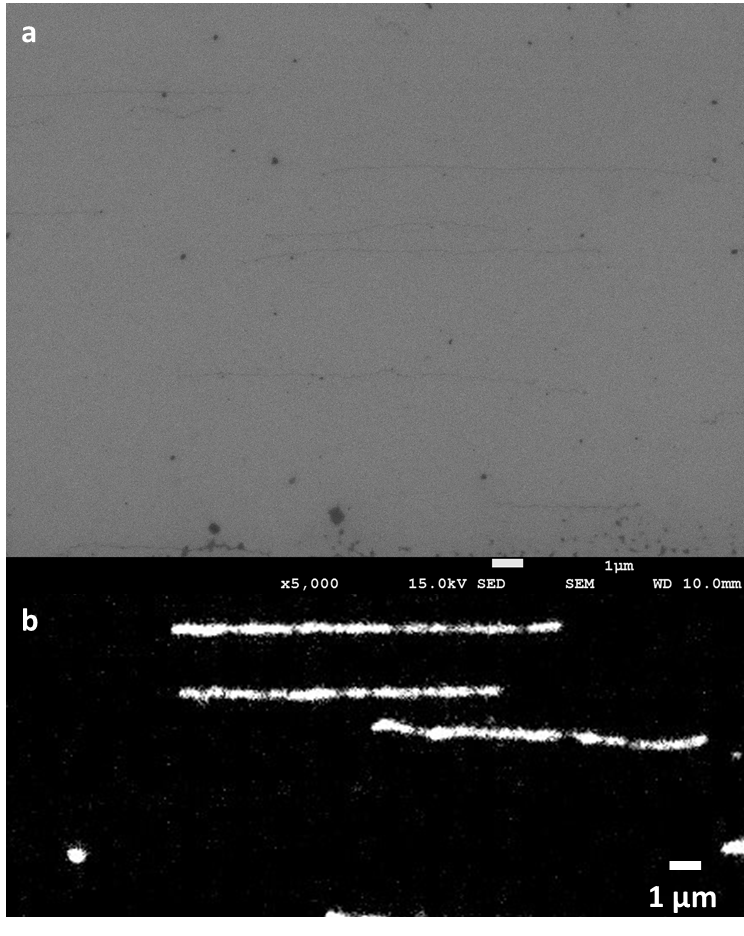


Fig. S5. H-NS-mScarlet and PVP stained DNA. a, 25 pg of λ DNA stained with H-NS-mScarlet (45 kDa, 264 nM) [7] and PVP (40 kDa, 5%). DNA molecules were elongated and immobilized onto a positively charged silicon wafer (coated with 1.1 mM Q-siloxane) inside a microfluidic device. The SEM image was acquired in SED mode at 15 kV, ×5,000 magnification. b, The corresponding FM image.


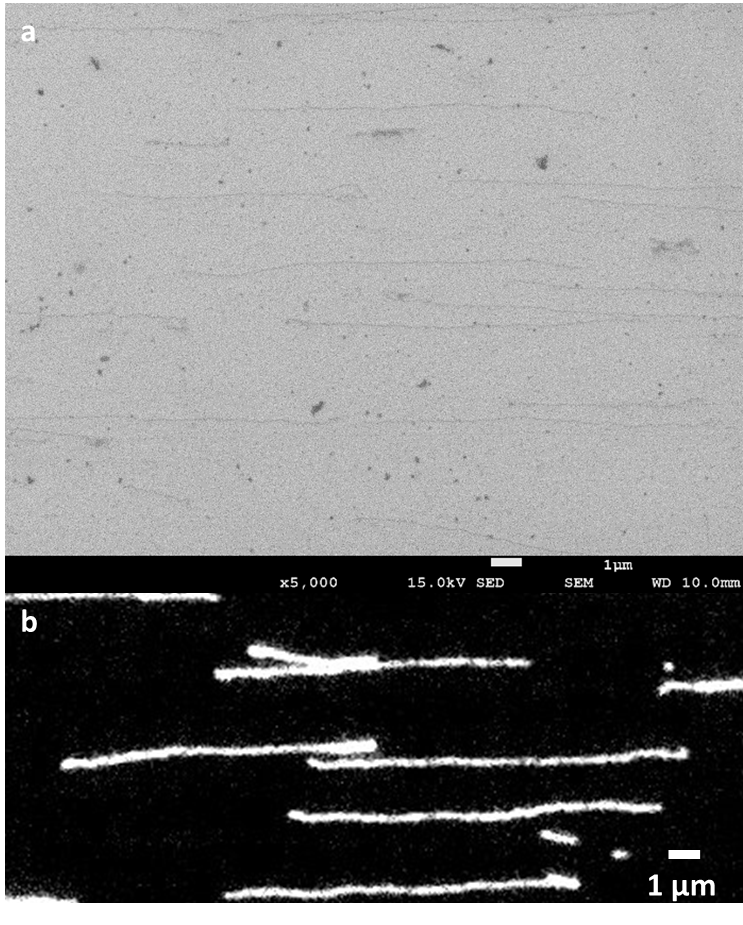


Fig. S6. tTALE-EmGFP and PVP stained DNA. a, 25 pg of λ DNA stained with tTALE-EmGFP (65 kDa, 396 nM) and PVP solution (40 kDa, 5%). DNA molecules were elongated and immobilized onto a positively charged silicon wafer (coated with 1.1 mM Q-siloxane) inside a microfluidic device. The SEM image was acquired in SED mode at 15 kV, ×5,000 magnification. b, The corresponding FM image.


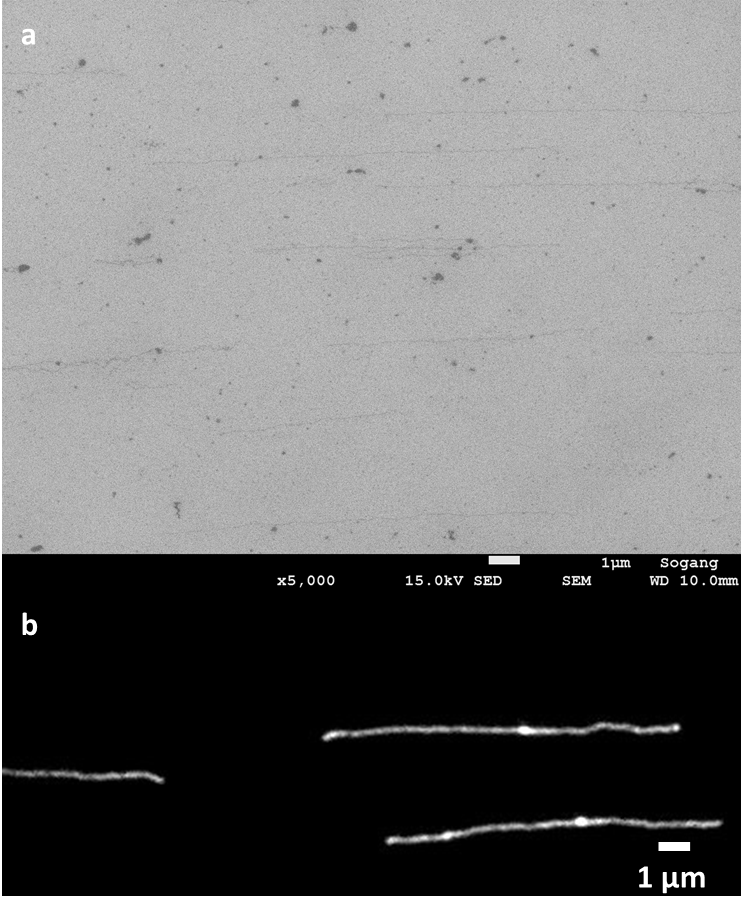


Fig. S7. Cro-mNeongreen and PVP stained DNA. a, 25 pg of λ DNA stained with Cro-mNeongreen (33 kDa, 264 nM) and PVP solution (40 kDa, 5%). DNA molecules were elongated and immobilized onto a positively charged silicon wafer (coated with 1.1 mM Q-siloxane) inside a microfluidic device. The SEM image was acquired in SED mode at 15 kV, ×5,000 magnification. b, The corresponding FM image.


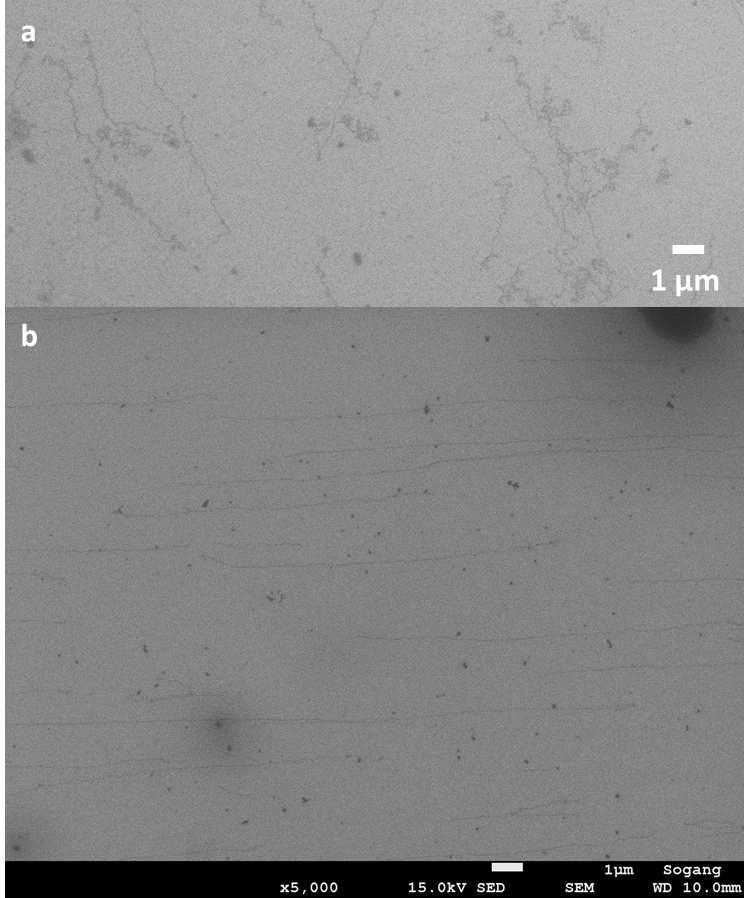


Fig. S8. poly(2-ethyl-2-oxazoline) and mNeongreen-HMG and stained DNA a, 2.5 ng of λ DNA stained with mNeongreen-HMG (31 kDa, 132 nM) and poly(2-ethyl-2-oxazoline) solution (50 kDa, 1% in water). DNA solution was deposited on positively charged silicon wafer (coated with 1.1 mM Q-siloxane) without microfluidic device. b, 25 pg λ DNA stained with mNeongreen-HMG (31 kDa, 132 nM) and poly(2-ethyl-2-oxazoline) solution (50 kDa, 1%) were elongated and immobilized onto a positively charged silicon wafer (coated with 1.1 mM Q-siloxane) inside a microfluidic channel. The SEM image was acquired in SED mode at 15 kV, ×5,000 magnification.


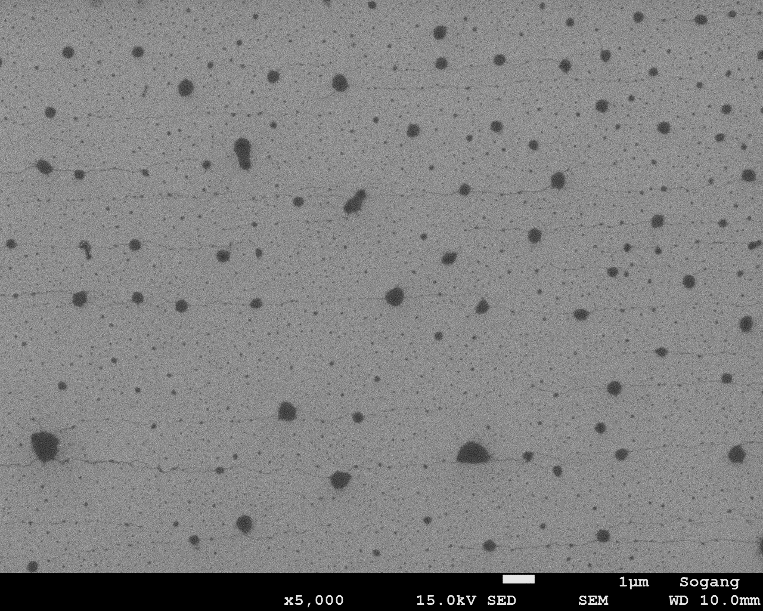


Fig. S9. Polyaniline and mNeongreen-HMG stained DNA. 25 pg of λ DNA stained with mNeongreen-HMG (31 kDa, 396 nM) and polyaniline solution (0.1% in water). DNA molecules were elongated and immobilized onto a positively charged silicon wafer (coated with 1.1 mM Q-siloxane) inside a microfluidic channel. The SEM image was acquired in SED mode at 15 kV, ×5,000 magnification.


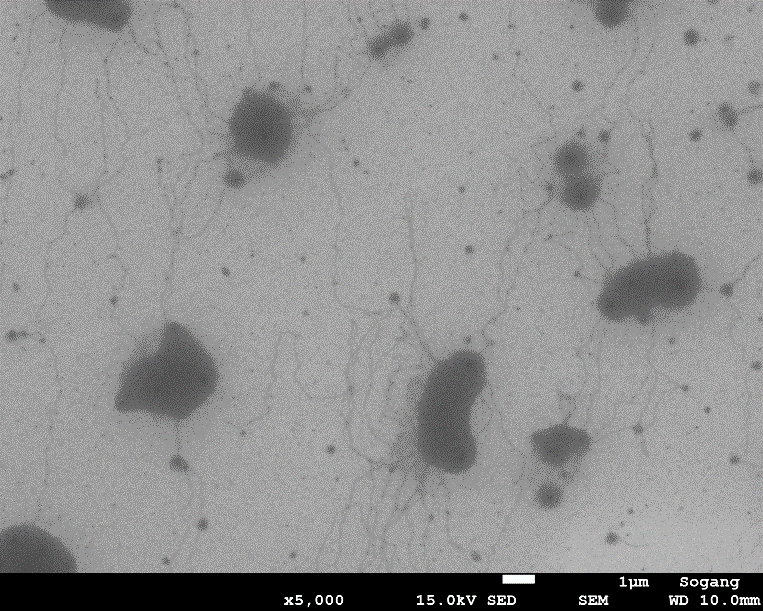


Fig. S10. Polyaniline and DNA mixture under SEM. 2.5 ng of λ DNA mixed with polyaniline solution (0.1%). DNA solution was deposited on positively charged silicon wafer (coated with 1.1 mM Q-siloxane) without microfluidic device. The SEM image was acquired in SED mode at 15 kV, ×5,000 magnification.

**References**

[1] a) E. Shin, W. Kim, S. Lee, J. Bae, S. Kim, W. Ko, H. S. Seo, S. Lim, H. S. Lee, K. Jo, *Sci Rep-Uk* **2019**, *9* (1), 17197, <https://doi.org/10.1038/s41598-019-53722-0>; b) Y. T. Kim, H. Oh, M. J. Seo, D. H. Lee, J. Shin, S. Bong, S. Heo, N. D. Hapsari, K. Jo, *Molecules* **2022**, *27* (16), <https://doi.org/10.3390/molecules27165248>.

[2] J. Park, S. Lee, N. Won, E. Shin, S.-H. Kim, M.-Y. Chun, J. Gu, G.-Y. Jung, K.-I. Lim, K. Jo, *Analyst* **2019**, *144* (3), 921, <https://doi.org/10.1039/C8AN01426D>.

[3] S. Bong, C. B. Park, S. G. Cho, J. Bae, N. D. Hapsari, X. L. Jin, S. Heo, J. E. Lee, K. Hashiya, T. Bando, H. Sugiyama, K. H. Jung, B. J. Sung, K. Jo, *Nucleic Acids Research* **2023**, <https://doi.org/10.1093/nar/gkad340>.

[4] Y. Kim, C. Noh, M. Y. H. Yu, M. Bae, K. Jo, *Chemical Communications* **2023**, *59* (61), 9388, <https://doi.org/10.1039/d3cc02112b>.

[5] a) T. Kim, S. Kim, C. Noh, H. Hwang, J. Shin, N. Won, S. Lee, D. Kim, Y. Jang, S. J. Hong, J. Park, S. J. Kim, S. Jang, K. I. Lim, K. Jo, *Talanta* **2023**, *252*, 123826, <https://doi.org/10.1016/j.talanta.2022.123826>; b) T. Kim, K. Jo, *Biochip Journal* **2023**, <https://doi.org/10.1007/s13206-023-00115-1>.

[6] X. L. Jin, N. D. Hapsari, S. Lee, K. Jo, *Analyst* **2020**, *145* (12), 4079, <https://doi.org/10.1039/d0an00218f>.

[7] a) S. Bong, C. B. Park, S. G. Cho, J. Bae, N. D. Hapsari, X. Jin, S. Heo, J. E. Lee, K. Hashiya, T. Bando, H. Sugiyama, K. H. Jung, B. J. Sung, K. Jo, *Nucleic Acids Res* **2023**, *51* (11), 5634, <https://doi.org/10.1093/nar/gkad340>; b) J. Park, S. Lee, N. Won, E. Shin, S. H. Kim, M. Y. Chun, J. Gu, G. Y. Jung, K. I. Lim, K. Jo, *Analyst* **2019**, *144* (3), 921, <https://doi.org/10.1039/c8an01426d>.
